# Effectiveness and safety profile of pembrolizumab for metastatic urothelial cancer: A retrospective single‐center analysis in Japan

**DOI:** 10.1002/cnr2.1398

**Published:** 2021-05-02

**Authors:** Motohiro Fujiwara, Takeshi Yuasa, Tetsuya Urasaki, Yoshinobu Komai, Ryo Fujiwara, Noboru Numao, Shinya Yamamoto, Junji Yonese

**Affiliations:** ^1^ Department of Urology Cancer Institute Hospital, Japanese Foundation for Cancer Research Tokyo Japan; ^2^ Department of Medical Oncology Cancer Institute Hospital, Japanese Foundation for Cancer Research Tokyo Japan

**Keywords:** immune checkpoint inhibitor, immune‐related adverse events, pembrolizumab, prognostic factor, urothelial carcinoma

## Abstract

**Background:**

The paradigm of medical treatment for metastatic urothelial carcinoma is dramatically changing through the introduction of pembrolizumab.

**Aim:**

We investigated the treatment effectiveness, the safety profile, and the prognostic factors of pembrolizumab in Japanese real‐world clinical practice.

**Methods and results:**

The medical records of 74 consecutive Japanese patients with metastatic urothelial cancer (UC), who started pembrolizumab as a second‐ or later‐line treatment at our institution between January 2018 and March 2020, were reviewed and statistically analyzed. The median follow‐up period after initiation of pembrolizumab was 8.5 (interquartile range: 3.5–15.7) months. The objective response rate was 30.2%, the median progression‐free survival period was 4.9 months, and the median overall survival (OS) period was 13.3 months. Evaluation revealed that 39 (52.9%) patients experienced adverse events (AEs), among whom eight patients (10.9%) had severe AEs (grade 3 or more), including grade 5 hemophagocytic syndrome. Multivariate analysis indicated that the presence of liver metastasis, worse performance status (≥2), elevated serum lactate dehydrogenase, and increased C‐reactive protein were predictive of shorter OS.

**Conclusion:**

We studied the effectiveness and safety profile of pembrolizumab therapy in Japanese UC patients. We believe that the data presented here will be useful for clinical physicians.

## 1 INTRODUCTION

Metastatic urothelial cancer (UC) is associated with a poor prognosis.^1^, ^2^ Platinum‐based chemotherapy has been the gold standard for this metastatic disease since 1985 and the introduction of methotrexate, vinblastine, adriamycin, cisplatin chemotherapy.^3^ No standard second‐line treatment has been established, consequently, metastatic UC is a generally fatal disease with overall survival (OS) of 6–7 months after the failure of first‐line chemotherapy.^4^ In December 2017, pembrolizumab (Keytruda, Merck), a highly selective, humanized monoclonal IgG4κ isotype antibody against programmed death 1 (PD‐1) that selectively inhibits the interaction between PD‐1 (which is expressed on activated T cells) and PD‐1 ligand 1 and 2 [which are expressed on antigen‐presenting cells and cancer cells] was approved as a second‐line treatment for use after platinum‐based chemotherapy for patients with metastatic UC in Japan.^1^, ^5^ Pembrolizumab have been approved for the treatment of metastatic UC that is not curatively resectable and has progressed after systemic chemotherapy including cisplatin. This greatly limits the proportion of all metastatic UC patients in which they are applicable, and even in those where applicable, the actual benefit to patients is not clearly established yet in terms of OS or quality of life improvements in the clinical practice.

Previously, we reported on our initial experience with pembrolizumab therapy in 2019,^6^ however, the data on actual clinical practice are still scarce. In this study, we investigated the treatment effectiveness, safety profile, and prognostic factors of pembrolizumab in Japanese real‐world clinical practice.

## 2 PATIENTS AND METHODS

### 2.1 Patients

The medical records of patients with previous platinum‐based chemotherapy for the treatment of metastatic UC who received pembrolizumab as second‐ or later‐line therapy at our institution between January 2018 and March 2020 were retrospectively reviewed. The study was approved by the approval of the institutional review board of the Cancer Institute Hospital, Japanese Foundation for Cancer Research and was performed in accordance with the guidelines of human research. All subjects involved in the study provided their written informed consent according to the Declaration of Helsinki.

### 2.2 Treatment and follow‐up examination

The primary endpoint of this study was the OS, whereas the progression‐free survival (PFS) period and objective response rate (ORR), defined as the sum of complete and partial response rates, and safety profile were analyzed as secondary endpoints. Pembrolizumab (200 mg) was administered every 3 weeks, as previously described.^1^, ^5^ We recorded the patients' medical history, including physical examination findings, Eastern Cooperative Oncology Group Performance Status (ECOG‐PS), laboratory findings, and chest radiography data before starting treatment and during pembrolizumab therapy, as assessed based on the attending physician's discretion. As the cut‐off value of laboratory findings, the upper limit of normal range (ULN) or lower limit of normal range was employed except for C‐reactive protein (CRP), hemoglobin (Hb), and the neutrophil‐lymphocyte ratio (NLR). According to the previous studies, 0.5 mg/dl, 10 g/dl, and 3.0 were employed as the cut‐off value of CRP, Hb, and NLR, respectively.^7^, ^8^, ^9^ The response to therapy was objectively evaluated by computed tomography every 2 or 3 months using the Response Evaluation Criteria in Solid Tumors guideline version 1.1.^10^ Toxicity was assessed by the Common Terminology Criteria for Adverse Events version 4.0.^11^

### 2.3 Statistical analysis

PFS periods, defined as the period from initial administration of pembrolizumab until the diagnosis of progressive disease, and OS period, defined as the period from initial administration of pembrolizumab to death from any cause, were assessed in all patients. Survival curves were estimated using the Kaplan‐Meier method. The risk factors of death of this study were analyzed using the Cox proportional hazard model. All statistical analyses were performed using JMP software version 12.2 (SAS Institute, Inc., Cary, NC) and *p*‐values <.05 were considered significant.

## 3 RESULTS

### 3.1 Patient characteristics

The study included 74 consecutive patients who were diagnosed with metastatic UC and commenced treatment with pembrolizumab after platinum‐based chemotherapy at our hospital. The characteristics of these patients are described in Table 1. Thirty‐eight (51.4%) had upper urinary tract UC and 36 patients (48.6%) had bladder UCs. Among these patients, 35 (47.3%) were administered pembrolizumab as the second‐line treatment, and 39 patients (52.7%) received it as the third‐ or fourth‐line treatment.

**TABLE 1:** Characteristics of patients treated with pembrolizumab (*n* = 74)

Characteristics	Median (IQR) or *n* (%)
Age (years)	69 (61–73)
Male/female	55 (74.3%)/19 (25.7%)
Pathological grade: 1 or 2/3	9 (12.2%)/65 (87.8%)
Primary tumor site	
Upper urinary tract (renal pelvis, ureter)	38 (51.4%)
Bladder	36 (48.6%)
ECOG PS ≥2 (%)	7 (9.5%)
Number of prior regimens: 1/2/3	35/33/6
Metastatic sites	
Lung/liver/lymph node/bone	31/9/57/13
Hemoglobin (g/dl)	11.5 (9.9–12.3)
CRP (mg/dl)	0.5 (0.1–2.9)
LDH (IU/L)	197 (169–230)
NLR	2.7 (1.9–4.1)
Bellmunt risks: 0, 1, 2, 3	28/36/9/1
Previous chemotherapy regimen	
Gemcitabine/cisplatin	43 (58.1%)
Gemcitabine/carboplatin	25 (33.8%)
Gemcitabine/cisplatin/etoposide	30 (40.5%)
Gemcitabine/cisplatin/paclitaxel	18 (24.3%)
Methotrexate/vinblastine/adriamycin/cisplatin	3 (4.1%)

*Note*: Bellmunt risk assesses the number of risk factors, which include worse performance status (>1), low hemoglobin (<10 mg/dl), the presence of liver metastasis, and short interval from previous chemotherapy (<3 months).

Abbreviations: CRP, C‐reactive protein; ECOG PS, Eastern Cooperative Oncology Group‐performance status; LDH, lactate dehydrogenase; NLR, neutrophil‐lymphocyte ratio.

### 3.2 Effectiveness of pembrolizumab

**FIGURE 1:**
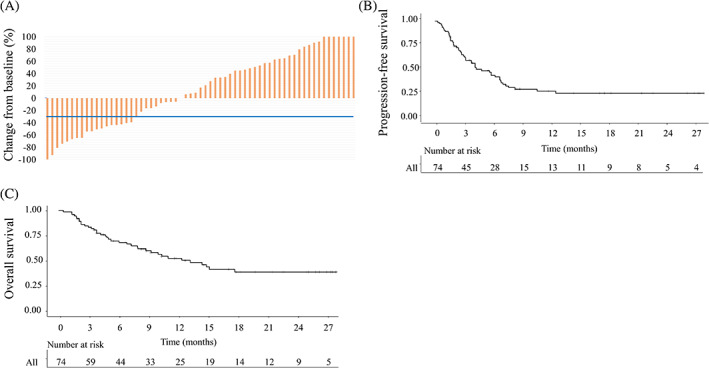
Effectiveness and prognostic factors of pembrolizumab treatment for patients with metastatic urothelial cancer. Waterfall plots of the response to pembrolizumab (A: *n* = 63). Progression‐free survival (B) and overall survival (C) curves (*n* = 74)

The median follow‐up period after pembrolizumab initiation was 8.5 months (interquartile range [IQR]: 3.5–15.7). Sixty‐three patients were evaluated for antitumor response. Among these patients, 50 (79.4%) showed decreased tumor size and the ORR was 30.2% (Figure 1A). Among the remaining 11 patients, three patients had no evaluable target lesion (small lymph node metastases), and eight patients showed clinical progression before the first imaging evaluation. In this study, all cases demonstrated a good response to pembrolizumab therapy demonstrated tumor shrinkage within 3 months after the start of pembrolizumab therapy, with no patient presenting initial tumor progression before shrinkage (pseudo‐progression). Fifty‐six patients (75.7%) stopped pembrolizumab therapy. The causes of discontinuation of pembrolizumab were disease progression (*n* = 45, 80.4%) and adverse events (AEs; *n* = 11, 19.6%). The remaining 18 patients (24.3%) were continuing this novel therapy at the time of writing. In addition, 36 patients (48.6%) died from disease progression and one patient (1.4%) died from immune‐related AEs (irAE; hemophagocytic syndrome). A total of 37 patients (50%) died during the study period. In addition, the estimated median PFS period was 4.9 months (IQR: 2.3 months—not reached) and the 3‐, 6‐, and 12‐month PFS rates were 64.9, 44.9, and 28.2%, respectively (Figure 1B). The median OS period was 13.3 months (IQR 3.3 months—not reached) and the estimated 6‐, 12‐ and 18‐month OS rates were 69.7, 52.3, and 38.7%, respectively (Figure 1C).

### 3.3 Adverse events

Thirty‐nine patients (52.9%) experienced AE, including eight (10.9%) with severe AE (grade 3 or more), as shown in Table 2. The most common AE was rash (*n* = 14, 18.9%), although none of them were of a severe grade. Eleven patients (14.9%) discontinued pembrolizumab therapy due to irAE. The AE that caused discontinuation were fatigue (*n* = 5, 6.8%), adrenal insufficiency (*n* = 3, 4.1%), interstitial pneumonia (*n* = 2, 2.7%), fulminant type1 diabetes mellitus (*n* = 1, 1.4%), and hemophagocytic syndrome (*n* = 1, 1.4%). Among the patients who experienced severe AE, one patient died due to grade 5 hemophagocytic syndrome, as described previously.^6^ This patient had polymyalgia rheumatica (PMR), which was being treated with 5 mg prednisolone. After the first administration of pembrolizumab, he complained of fatigue and joint stiffness, which was diagnosed as an exacerbation of PMR. Although he discontinued pembrolizumab after the first administration, his platelet count decreased (19 000/mm^3^) without any other abnormal signs (PS = 0) 2 months later. We immediately commenced steroid pulse therapy (1000 mg for 3 days) followed by intravenous immune globulin, cyclosporine administration, and plasma exchange, with appropriate antibiotic and transfusion support. However, he died of multiple organ dysfunction 13 days after admission.

**TABLE 2:** Adverse events (AE) of patients treated with pembrolizumab (*n* = 74)

	G1‐2 (*n*)	%	G3‐5 (*n*)	%	Total (%)
Number of patients with AEs	31	42.0	8	10.9	39 (52.9)
Types of AEs					
Rash	14	18.9	0	0	14 (18.9)
Hypothyroidism	9	12.2	0	0	9 (12.2)
Adrenal insufficiency	2	2.7	3	4.1	5 (6.8)
Interstitial pneumonia	3	4.1	2	2.7	5 (6.8)
Diarrhea	1	1.4	0	0	1 (1.4)
Dysgeusia	2	2.7	0	0	2 (2.7)
Hyperglycemia	0	0	2	2.7	2 (2.7)
Hemophagocytic syndrome	0	0	1	1.4	1 (1.4)

### 3.4 Risk factors for short survival period

Finally, we investigated the variables that could predict a shorter OS period for patients with metastatic UC treated with pembrolizumab as second‐ or later‐line therapy. On univariate analysis, worse ECOG‐PS (≥2), the presence of liver metastasis, the presence of bone metastasis, lower Hb, high serum lactate dehydrogenase (LDH) levels (≥ULN), and high serum CRP levels were extracted as factors predictive of a shorter OS as shown in Table 3. On multivariate analysis, worse ECOG‐PS (≥2) (hazard ratio [HR]: 6.0, *p* = .007), the presence of liver metastasis (HR: 14.4, *p* < .001), high LDH (HR: 3.0, *p* = .005), and higher CRP (HR: 2.4, *p* = .018) were found to be independent factors predictive of a shorter OS period (Table 3).

**TABLE 3:** Poor prognostic factors for overall survival of the patients treated pembrolizumab (*N* = 74)

	Univariate analysis			Multivariate analysis		
Variables	*p*‐Value	Hazard ratio	95% CI	*p*‐Value	Hazard ratio	95% CI
Female	0.055	2.041	0.985–4.003			
Age > 70 years old	0.599	0.832	0.403–1.626			
High ECOG performance status (≥2)	<0.001	11.723	3.679–36.505	0.007	5.962	1.645–21.601
Upper tract urothelial carcinoma	0.639	1.168	0.609–2.246			
Surgical treatment	0.068	0.541	0.283–1.047			
Lung metastasis	0.467	1.274	0.658–2.438			
Liver metastasis	<0.001	10.389	4.353–22.970	<0.001	14.359	4.923–40.265
Bone metastasis	<0.001	3.832	1.827–7.634	0.801		
Low hemoglobin (<10 mg/dl)	0.013	2.525	1.208–5.933	0.075		
Elevated serum LDH (>ULN)	0.006	2.743	1.358–5.363	0.005	3.035	1.427–6.337
Elevated serum CRP (>0.5 mg/dl)	0.009	2.422	1.250–4.912	0.018	2.431	1.161–5.376
Increased NLR (>3)	0.622	1.179	0.605–2.253			
Increased platelet count (>400 000)	0.364	1.493	0.598–3.245			

Abbreviations: 95% CI, 95% confidence interval; CRP, C‐reactive protein; ECOG, Eastern Cooperative Oncology Group; LDH, lactate dehydrogenase; NLR, neutrophil‐lymphocyte ratio.

## 4 DISCUSSION

In this study, we elucidated the effectiveness and safety profile of pembrolizumab in Japanese patients with metastatic UC. The ORR was 30.2% and the estimated median PFS and OS periods were 4.9 and 13.3 months, respectively (Figure 1A–C). Six, 12‐ and 24‐month OS rates were 69.7, 52.3, and 38.7%, respectively. All these results are comparable with or slightly better than those reported in the international phase III the KEYNOTE‐045 trial.^5^

In the present study, 52.7% of patients experienced irAE, including 21.6% who experienced grade 3 or higher irAE. These results are similar to the findings of the pivotal KEYNOTE‐045 trial.^5^ The most common AE was rash of less than or equal to grade 2 (19%). In our study, one patient, who had PMR, an autoimmune disease, died due to grade 5 hemophagocytic syndrome, which we reported previously.^6^ Hemophagocytic syndrome triggered by immune checkpoint inhibitors is not entirely clear. Although rare and most cases were reported to be manageable with standard treatment algorithms, we must be aware that it is a potentially fatal irAE of pembrolizumab.^6^, ^12^

Clinical trials, including the KEYNOTE‐045 study, usually exclude patients with pre‐existing autoimmune diseases. Thus, the effectiveness and safety profile of immune checkpoint inhibitors in patients with pre‐existing autoimmune diseases have been the subject of clinical studies.^13^, ^14^ A multi‐institutional retrospective study from Italy reported that the incidence of irAE in the patients with pre‐existing autoimmune diseases (*n* = 85) was significantly higher than that in the patients without autoimmune diseases (*n* = 66; 65.9 vs 39.9%).^13^ This study also reported that pre‐existing autoimmune diseases were not significantly related to PFS and OS.^13^ On the other hand, a multi‐institutional study from France (*n* = 112) demonstrated relatively better effectiveness (ORR: 49%) than expected compared with patients without autoimmune diseases.^14^ This study also reported that flares (47%) or irAE (42%) occurred frequently in these patients in spite of mostly manageable irAE without discontinuation.^14^ This important medical theme was well‐reviewed by Boland et al.^15^ Further careful investigations are necessary to determine an effective immune‐checkpoint inhibitor therapy that does not cause the flare‐up of symptoms in patients with pre‐existing autoimmune disease.

In this study, the presence of liver metastasis, worse PS (≥2), higher LDH levels (>ULN), and higher CRP levels (>0.5 mg/dl) were extracted as independent predictors of a poor prognosis for patients with advanced UC receiving pembrolizumab. Previously, in the platinum‐therapy era, several prognostic factors were reported to predict the prognosis of patients with UC. Bajorin et al. found that a Karnofsky performance status <80% and the presence of visceral (liver, lung, and bone) metastasis were independent prognostic factors associated with the OS.^16^ Bellmunt et al. also reported that liver metastasis, poor PS (≥2), and Hb less than 10 mg/dl were poor prognostic factors in metastatic UC patients after platinum‐based chemotherapy.^8^ In the phase 3 trial KEYNOTE‐045 study, the ECOG‐PS score (≥2), the presence of liver metastases, Hb (<10 g/dl), and time from the last dose of chemotherapy (3 months) were used as stratification factors.^5^

The presence of higher LDH levels has also been previously reported to be a poor prognostic factor for metastatic UC.^17^ Lactate and pyruvate are interconvertible via the enzymatic action of LDH. Elevated LDH induces overexpression of hypoxia, which stimulates the vascular endothelial growth factor and aggressive angiogenesis.^18^ Therefore, angiogenesis, which was stimulated by elevated LDH, can be considered to have an impact on the survival of patients with metastatic UC. The elevation of CRP levels, which is a representative acute phase reactant that is widely used to evaluate systemic inflammation, is also known to be a poor predictor of advanced UC, as described previously.^19^, ^20^ The increasing CRP level reflects tumors capable of producing significant amounts of proinflammatory cytokines—in particular, interleukin‐6.^20^ Accordingly, the presence of liver metastasis, worse PS (≥2), higher LDH levels (>ULN), and higher CRP levels (0.5 mg/ml), which were extracted as poor prognostic factors in this study, can be considered as metastatic UC‐related poor prognostic factors treated with pembrolizumab therapy. These factors, which are easily and cheaply obtained biomarkers, can provide helpful information to predict the outcome in metastatic UC patients treated with pembrolizumab.

Several limitations exist in the current study. First, this is a retrospective small study in a single institution. Therefore, there was a possible bias in extracting the prognostic factors. Thus, external validation is essential before implementing our findings in clinical practice. Second, scheduled imaging studies, which are common examinations in clinical trials, were not performed. Thus, a simple comparison between our results and those of clinical trials might be difficult.

In conclusion, we studied the effectiveness and safety profile of pembrolizumab therapy in Japanese UC patients. The ORR was 30.2% and the 12‐ and 24‐month OS rates were 52.3, and 38.7%, respectively. We believe that the data presented here will be useful for clinical physicians.

## CONFLICT OF INTEREST

T.Y received remuneration for lectures from MSD Japan (Tokyo, Japan). The other authors declare that they have no conflicts of interest that might be relevant to the contents of this manuscript.

## AUTHOR CONTRIBUTIONS

**T.Y:** Conceptualization; project administration; supervision. **T.U:** Supervision. **Y.K:** Supervision. **R.F:** Supervision. **N.N:** Supervision. **S.Y:** Supervision. **J.Y:** Supervision.

## ETHICS STATEMENT

The study was approved by the approval of the institutional review board of the Cancer Institute Hospital, Japanese Foundation for Cancer Research and was performed in accordance with the guidelines of human research. All subjects involved in the study provided their written informed consent according to the Declaration of Helsinki.

## Data Availability

Research data are not shared.
